# Pediatric hyperuricemia: genetic basis, metabolic mechanisms, and clinical implications

**DOI:** 10.3389/fendo.2026.1841803

**Published:** 2026-08-10

**Authors:** Jing Zhou, Jianing Lv, Chunhuan Hong, Qinhao Liu, Shuqing Jin, Yi Zhang, Yunfeng Liu

**Affiliations:** 1 Department of Endocrinology, First Hospital of Shanxi Medical University, Taiyuan, China; 2 First Clinical Medical College, Shanxi Medical University, Taiyuan, China; 3 Department of Pharmacology, Shanxi Medical University, Taiyuan, China; 4 Medicinal Basic Research Innovation Center of Chronic Kidney Disease, Ministry of Education, Shanxi Medical University, Taiyuan, China

**Keywords:** children and adolescents, chronic kidney disease, obesity, pediatric hyperuricemia, urate-lowering therapy, uric acid

## Abstract

Hyperuricemia (HUA) is a heterogeneous group of metabolic disorders caused by long-term disturbances in purine metabolism. In recent years, although adult HUA and gout have been extensively studied, the understanding of HUA and gout in children and adolescents remains insufficient. Diagnostic criteria for adult HUA are well-established, but no consensus has yet been reached regarding its definition in children and adolescents. This review summarizes the current understanding of pediatric HUA, emphasizing its genetic basis, metabolic mechanisms, and clinical associations. ATP-binding cassette subfamily G member 2 (ABCG2) dysfunction, gene-defined forms of autosomal dominant tubulointerstitial kidney disease (ADTKD), particularly ADTKD-UMOD and ADTKD-REN, and selected purine metabolism disorders contribute to early-onset HUA, gout, nephrolithiasis, and renal involvement. Obesity-related insulin resistance (IR) may promote urate accumulation through pathways linked to oxidative stress, endothelial dysfunction, and inflammasome activation. Persistent elevation of uric acid (UA) has been associated with chronic kidney disease (CKD) progression, elevated blood pressure (BP), and subclinical cardiovascular remodeling. However, many of these mechanistic associations are supported primarily by adult and experimental studies and require further validation in children. We also review non-pharmacological management and indication-based pharmacological treatment strategies for pediatric HUA. These indications include asymptomatic hyperuricemia (AH), pediatric gout, UA nephrolithiasis, inherited purine metabolism disorders, CKD-associated HUA, and tumor lysis syndrome (TLS)-associated acute HUA, with emphasis on pediatric evidence, regulatory approval, off-label use, dosing, monitoring, and safety. In children, asymptomatic serum urate elevation is often the first recognized presentation, whereas nephrolithiasis, gout, renal dysfunction, or acute HUA should prompt evaluation for genetic, metabolic, renal, or treatment-related causes. These findings underscore the need for early identification and personalized intervention in children with HUA to prevent long-term renal and metabolic complications.

## 1 Introduction

Hyperuricemia (HUA) is a common metabolic condition characterized by an excessive increase in serum uric acid (SUA) levels due to impaired purine metabolism or decreased uric acid (UA) excretion from the body. It has shown a trend of younger age and a significant increase in incidence. The proposed definition of HUA is a SUA level of >7.0 mg/dL in men or >5.7 mg/dL in women (1). There is still no unanimity on how to define HUA in children and adolescents. However, SUA levels in children fluctuate with age. They are quite low in the newborn period but rise to adult levels after puberty (2). Childhood HUA is associated with the occurrence and progression of various diseases, including obesity, kidney diseases, cardiovascular diseases (CVDs), metabolic syndrome (MetS), impaired glucose metabolism, and genetic diseases. Moreover, it is associated with a higher risk of early organ damage compared with adults. A recent study demonstrated that higher UA levels in childhood are significantly associated with an increased risk of high blood pressure (BP) in young adulthood (3). In this context, it is clearly very important to effectively prevent and treat HUA starting from childhood through adolescence, which requires age-stratified diagnosis and personalized interventions to reduce lifelong health risks. This review aims to concisely and critically summarize the current understanding of pediatric HUA and to discuss the available treatment options.

## 2 Reference values of uric acid in children and adolescents

In adults, HUA has been defined in NHANES as an SUA level >7.0 mg/dL in men and >5.7 mg/dL in women (1). UA levels in children and adolescents vary at different stages of development. The SUA levels in children show a gradual increase from infancy to adolescence, with the most rapid rise occurring during adolescence (2). The SUA levels in males rise more rapidly than in females post-puberty, while a more pronounced increase is observed in females following menopause. The definition of HUA in children needs to take into account factors including age, sex, and pubertal development. A global consensus has not yet been established regarding the diagnostic criteria for HUA in children. The prevailing literature primarily adopts the cutoff threshold established by the Mayo Clinic (4). Nevertheless, these reference intervals were generated from specific regional populations and may be affected by differences in ethnicity, dietary patterns, and lifestyle. As a result, caution is warranted when extrapolating these criteria to pediatric populations worldwide. **Table 1** provides a descriptive summary of published SUA distributions across different pediatric populations and should not be interpreted as a set of validated diagnostic reference intervals.

**Table 1:** Published pediatric serum uric acid distributions and study-specific hyperuricemia thresholds stratified by age and sex where available.

Reference	Value type	Sex	Age (years)	Serum uric acid value/cut-off (mg/dL)
Kubota (198)	Hyperuricemia cut-off, mean + 2 SD	Both sexes	<1	4.6
		Both sexes	1-3	4.8
		Both sexes	4-6	5.5
		Both sexes	7-9	5.9
		Both sexes	10-12	6.1
		Male	13-15	7.0
		Female	13-15	6.2
Noone (199)	Upper limit of normal reference range	Both sexes	<1	8.4
		Both sexes	1-10	5.4
		Male	11-15	7.9
		Female	11-15	5.9
Lee (200)	Hyperuricemia definition	Both sexes	10-11	>6.6
		Male	12-18	>7.7
		Female	12-18	>5.7
Moulin-Mares (201)	P90 upper reference value	Male	6-9	4.5
		Male	10-13	5.7
		Male	14-17	6.4
		Female	6-9	4.8
		Female	10-13	5.2
		Female	14-17	5.3
Ye (202)	Age-/sex-specific 95% reference interval; 97.5th percentile upper reference value	Both sexes	2-5	6.8
		Both sexes	6-9	6.9
		Male	10-11	7.5
		Male	12-13	9.2
		Male	14-18	9.8
		Female	10-11	7.1
		Female	12-13	7.2
		Female	14-18	7.3

All values are reported as serum uric acid concentrations in mg/dL. Because the included studies differ in population, study design, age/sex stratification, pubertal assessment, and threshold definition, this table should be interpreted as a descriptive summary of published pediatric serum uric acid distributions or study-specific hyperuricemia thresholds rather than as a unified pediatric reference interval.

## 3 Physiological effects of uric acid

UA, the terminal product of purine catabolism, exists primarily as urate at physiological pH (5). As the concentration of urate in the blood increases, the formation of UA crystals also increases. When urate levels exceed 6.8 mg/dL (400 μmol/L), the limit of solubility under physiological conditions, crystals of monosodium urate (MSU) may form (1). Approximately two-thirds of urate is eliminated by the kidneys, with the remainder excreted in the stool (5). Urate influences humans through several mechanisms, including antioxidant, pro-oxidant, pro-inflammatory, nitric oxide (NO) regulation, and immune system interactions (6–9).

## 4 Genetic and molecular mechanisms

### 4.1 ATP-binding cassette subfamily G member 2 and pediatric-onset hyperuricemia and gout

Genome-wide association studies (GWAS) have consistently identified the ATP-binding cassette subfamily G member 2 (ABCG2) locus as a significant genetic determinant of gout susceptibility (10). The p.Q141K variant in ABCG2 serves as a strong and independent genetic risk factor for childhood-onset HUA and gout (11). A study of 705 Japanese patients with gout reported that 88.2% of early-onset cases (twenties or younger) carried mild-to-severe ABCG2 dysfunction variants (12). Consistently, in a Central European cohort, non-synonymous ABCG2 variants were found in 7 of 8 patients with gout onset between 10 and 20 years, with p.Q141K present in 6 of these variant carriers (13). Mechanistically, loss-of-function variants in ABCG2 impair extra-renal urate excretion and consequently predispose individuals to HUA. These variants also influence the therapeutic response to urate-lowering therapy (ULT). Febuxostat can potently inhibit ABCG2, potentially reducing ABCG2-mediated urate excretion and partially offsetting its serum urate-lowering effect (14).

### 4.2 Gene-based ADTKD subtypes and pediatric hyperuricemia

Autosomal dominant tubulointerstitial kidney disease (ADTKD) is a rare inherited kidney disorder caused by pathogenic variants in several genes, including UMOD, MUC1, and REN, with less common or syndromic forms involving HNF1B and SEC61A1 (15–19). Familial juvenile hyperuricemic nephropathy (FJHN), medullary cystic kidney disease (MCKD), and uromodulin-associated kidney disease (UAKD) are historical or partially overlapping terms that are no longer preferred as primary diagnostic labels. To reduce terminological ambiguity and improve diagnostic consistency, the KDIGO consensus recommended a gene-based nomenclature using “ADTKD” followed by the causative gene, such as ADTKD-UMOD, ADTKD-REN, and ADTKD-MUC1 (15).

Among the genetic causes of pediatric HUA, ADTKD-UMOD and ADTKD-REN are of particular clinical relevance. ADTKD-UMOD is usually caused by missense variants in UMOD, which lead to misfolding of mutant uromodulin and its retention in the endoplasmic reticulum of thick ascending limb cells (17). This impairs uromodulin trafficking and disturbs tubular handling of sodium and urate. Consequently, it leads to a decrease in the fractional excretion of urate, the early onset of HUA, and, in certain patients, the development of gout during adolescence or early adulthood (20, 21). In the Wake Forest cohort, 34 children with ADTKD-UMOD were younger than 18 years. At initial evaluation, only 21% had an estimated glomerular filtration rate (eGFR) above 90 mL/min/1.73 m², while 44% had chronic kidney disease (CKD) stage 2 and 32% had CKD stage 3 (22). Although this referral-center cohort may be subject to selection bias, these findings suggest that impaired kidney function is not uncommon among children with HUA in the context of ADTKD-UMOD. Moreover, the prevalence of CKD tends to rise as these children grow older. Overall, end-stage kidney disease (ESKD) in ADTKD-UMOD usually develops in adulthood, with a median age of 47 years and uncommon occurrence before age 30 (23). Therefore, the main clinical priorities in childhood are early diagnosis, family screening, genetic counseling, and long-term renal follow-up, rather than short-term prediction of progression to ESKD.

ADTKD-REN is a relatively uncommon disorder. Nevertheless, it should be considered in children with otherwise unexplained HUA accompanied by early kidney dysfunction, anemia disproportionate to the degree of CKD, mild hyperkalemia, metabolic acidosis, low or low-normal renin levels, or a family history compatible with autosomal dominant inheritance (22, 24). This childhood/adolescent-onset presentation is most often associated with REN variants affecting the signal peptide or prosegment of preprorenin. In contrast, variants within the mature renin peptide typically result in a milder clinical phenotype with a later onset, which is commonly characterized by the development of gout in adulthood or a gradually progressive course of CKD (24, 25).

In contrast, ADTKD-MUC1 and HNF1B-related disease are less directly linked to pediatric HUA. ADTKD-MUC1 is primarily characterized by progressive tubulointerstitial CKD with bland urinary sediment and usually lacks distinctive clinical or biochemical features during childhood (26, 27). HUA and occasional gout in ADTKD-MUC1 generally occur in the setting of progressive renal insufficiency rather than as primary early manifestations. Moreover, renal failure most commonly ensues during adulthood in affected individuals (26, 28, 29). Therefore, the main pediatric relevance of ADTKD-MUC1 is the differential diagnosis of unexplained autosomal dominant CKD. HNF1B-related disease is usually recognized through renal developmental or cystic abnormalities, electrolyte disturbances, pancreatic or hepatic involvement, or familial kidney disease and diabetes. Although HUA has been reported in pediatric patients, it is rarely the initial diagnostic clue (15, 22).

In addition, preliminary evidence suggests that rare variants in SLC8A1 may act as genetic modifiers of UMOD-related disease severity. However, current data are insufficient to classify SLC8A1 as an established independent causative gene for ADTKD or pediatric HUA (30).

### 4.3 Genetic syndromes and purine disorders

Disorders of purine metabolism result from enzymatic abnormalities or dysfunctions that impair the synthesis, degradation, or excretion of purine nucleotides. These disruptions may result in HUA and associated consequences, including gout, nephrolithiasis, and neurological deficits (31). Notable disorders include complete hypoxanthine-guanine phosphoribosyltransferase (HGPRT) deficiency (Lesch-Nyhan syndrome), adenine phosphoribosyltransferase (APRT) deficiency, phosphoribosyl pyrophosphate (PRPP) synthetase hyperactivity, and glycogen storage diseases. The lack of HGPRT is the primary cause of Lesch–Nyhan syndrome (LNS). HGPRT deficiency results in reduced purine recycling, hence increasing purine breakdown. This degradation underlies the peripheral HUA in LNS patients (32). Glycogen storage disease type I (GSD I) is an important inherited metabolic cause of pediatric HUA. It is caused by defects in glucose-6-phosphatase activity or glucose-6-phosphate transport. The disease typically manifests clinically during infancy, characterized by a cluster of symptoms such as hepatomegaly, fasting hypoglycemia, lactic acidemia, hyperlipidemia, and HUA (20, 33).

Down syndrome provides another example of age-dependent purine dysregulation. In a single-center study of 238 children and adolescents with Down syndrome, SUA levels were higher than those in the general pediatric population across all age groups, and HUA cases increased rapidly after 10 years of age (34). As additional biochemical support, overexpression of the CBS gene, which encodes cystathionine β-synthase, has been proposed to favor flux through the homocysteine (Hcy) transsulfuration pathway. Adenosine, generated via S-adenosylhomocysteine breakdown, is ultimately converted to UA. However, clinical evidence in children and young adults is limited to association: in a cohort of 147 individuals with Down syndrome, Hcy and UA levels increased with age and showed a moderate positive correlation after age adjustment (35).

## 5 Diseases associated with hyperuricemia

### 5.1 Obesity

In children, obesity and HUA have been strongly correlated, according to numerous epidemiological studies. A cross-sectional study showed that the overall estimated prevalence of HUA in obese children was 54.8%, with a gradual increase observed over the past seven years (36). In a longitudinal cohort study spanning childhood to adulthood, Yun et al. examined the temporal relationship between body mass index (BMI) and UA and discovered that increases in BMI preceded changes in UA, supporting a temporal and potentially causal relationship between higher BMI and elevated UA levels (37). A Mendelian randomization analysis further supported this directionality (38).

Besides BMI, which has been associated with increased HUA risk, additional obesity indicators such as skeletal muscle (SM), skeletal muscle mass (SMM), hip circumference (HC), body fat mass (BFM), waist circumference (WC), and waist-to-height ratio (WHtR) also play a role in the development of HUA in children (39, 40). Xie et al. reported that SMM and the percentage of skeletal muscle (PSM), assessed by bioelectrical impedance analysis, were more strongly associated with the risk of HUA than BFM and waist or HC in obese children and adolescents (39). These findings suggest that both adiposity and lean-mass-related metabolic changes may contribute to urate dysregulation in obese children. Proposed explanations include increased purine turnover related to greater muscle mass and reduced renal urate excretion associated with obesity-related insulin resistance (IR), although these mechanisms require further pediatric validation.

Several mechanistic studies provide biological plausibility for the observed association between obesity and HUA, although the current evidence mainly comes from adults, animals, and *in vitro* experiments. Experimental evidence suggests that hypoxia can increase xanthine dehydrogenase/xanthine oxidase (XDH/XO) activity and oxidative injury in endothelial cells (41). In obese mouse models and adipocyte experiments, adipose tissue has also been shown to produce and secrete UA through xanthine oxidoreductase (XOR), with this process being enhanced under obesity- or hypoxia-related conditions (42). In addition, studies in obese adipose tissue and adipocyte models indicate that local hypoxia may disrupt adipokine production, promote inflammatory responses, impair insulin signaling, and alter lipid metabolism (43, 44). IR, a common consequence of obesity, contributes to high SUA levels. The reason for this was that IR can enhance the reabsorption of UA in the proximal tubule of the kidney, lowering UA excretion and leading to the development of HUA (45, 46). HUA may be associated with the processes of lipogenesis and lipolysis. Activation of the pentose phosphate pathway during lipogenesis enhances purine synthesis and triglyceride storage, both contributing to elevated SUA levels (42). Overall, these experimental and metabolic findings provide mechanistic plausibility for obesity-related HUA, but direct pediatric evidence remains sparse. In children and adolescents, available studies mainly show that elevated SUA is associated with obesity, IR, dyslipidemia, and adipokine abnormalities (47), whereas the roles of adipose XOR activation, lipid–purine flux, and renal urate handling require further validation.

### 5.2 Insulin resistance, impaired glucose metabolism, and diabetes risk

Pediatric observational evidence indicates that, in children and adolescents, elevated SUA is more consistently associated with IR and impaired glucose metabolism than with overt diabetes itself (48, 49). In contrast, adult prospective studies have shown that higher SUA levels are associated with incident type 2 diabetes (50). However, pediatric investigations directly assessing HUA as a predictive marker for diabetes are notably limited. A study of 134 obese children and adolescents and 111 normal-weight children and adolescents found that each 1 mg/dL increase in SUA levels was associated with 91% higher odds of IR (49). In children with CKD, SUA was positively correlated with homeostasis model assessment of insulin resistance (HOMA-IR), but the association appeared to be largely mediated by relative fat mass (51). Niu Y. et al. discovered that UA had an indirect impact on the relationship between BMI and homeostasis model assessment of insulin resistance (HOMA-IR), while no association was observed with fasting blood glucose (52). A cross-sectional study involving 2,248 overweight or obese (OW/OB) youths aged 5–17 years found that the prevalence of impaired glucose tolerance (IGT) and reduced insulin sensitivity (IS) increased progressively across quintiles of UA, while impaired fasting glucose (IFG) and IR were only associated with the highest quintile (48). A targeted metabolomic analysis of obese children aged 12 to 18 years showed a significant positive association between HOMA-IR and metabolic variables such as UA levels in boys but not in girls (53). This finding suggests that sex-related differences may influence the development of pubertal IR. The temporal and causal relationship between elevated SUA, IR, and progression to diabetes in pediatric populations remains unresolved.The biological mechanisms linking HUA to impaired glucose metabolism remain incompletely understood, and direct pediatric mechanistic evidence is limited. HUA has been shown to directly impair pancreatic β-cell function, leading to defective insulin secretion and a reduction in functional β-cell mass (54). Elevated UA may impair endothelial insulin signaling and reduce NO bioavailability, thereby contributing to endothelial dysfunction and systemic IR (55). Moreover, increased UA levels are closely associated with inflammatory responses, activating the NOD-like receptor family pyrin domain-containing 3 (NLRP3) inflammasome and triggering the release of proinflammatory cytokines that disrupt glucose metabolism (56). Consistently, adult observational evidence links chronic low-grade inflammation to IR, with elevated hsCRP independently associated with reduced IS (57). At the molecular level, oxidative stress represents a central pathway through which UA drives IR (42). Increased SUA promotes the conversion of XDH to XO and activates nicotinamide adenine dinucleotide phosphate oxidase (NOX) and hypoxia-inducible factor-1α (HIF-1α), leading to excessive intracellular generation of reactive oxygen species (ROS) (58, 59). Among the downstream targets of ROS signaling, thioredoxin-interacting protein (TXNIP) has emerged as a pivotal mediator. Upregulation of TXNIP by UA induces mitochondrial reactive oxygen species (MtROS) overproduction and suppresses phosphorylation of the insulin receptor substrate 2 (IRS2)/protein kinase B (AKT) signaling cascade, thereby impairing insulin signaling in macrophages. Notably, UA-induced oxidative stress also activates the TXNIP-dependent nuclear factor erythroid 2–related factor 2 (Nrf2)/heme oxygenase-1 (HO-1) pathway, whereas inhibition or silencing of TXNIP has been shown to markedly attenuate UA-mediated oxidative stress and IR (60).

### 5.3 Metabolic syndrome

Goli et al.’s meta-analysis confirmed a significant association between elevated SUA and individual MetS components in children, including fasting glucose, fasting insulin, triglycerides, HDL-C, WC, and BP (61). Cho et al. analyzed data from 1,349 Korean children and adolescents aged 10–19 years from the KNHANES 2016–2017 survey and found that higher SUA quartiles were associated with increased odds of MetS in children and adolescents, particularly in relation to abdominal obesity and low HDL-C (62).

Mechanistic evidence from adult studies, animal models, and *in vitro* experiments suggests that UA may contribute to MetS through reduced NO bioavailability, oxidative stress, adipose inflammation, and impaired insulin signaling (58, 63, 64). Therefore, in children and adolescents, SUA may be better interpreted as a potential integrative marker of cardiometabolic risk rather than as a proven causal driver of MetS.

### 5.4 Metabolic dysfunction-associated steatotic liver disease

According to the 2023 multisociety Delphi consensus on steatotic liver disease nomenclature, the term nonalcoholic fatty liver disease (NAFLD) has been replaced by metabolic dysfunction-associated steatotic liver disease (MASLD), and nonalcoholic steatohepatitis (NASH) by metabolic dysfunction-associated steatohepatitis (MASH) (65). Most studies cited in this section predated the nomenclature revision; therefore, findings reported under NAFLD/NASH are interpreted within the current MASLD/MASH framework.

MASLD is the hepatic manifestation of MetS, and multiple pediatric observational studies report strong associations with HUA (66, 67). Children with obesity and HUA are more prone to MASH independently of other factors (68). Nobili et al. discovered that in children with MASLD, higher SUA levels were linked to steatosis and fibrosis in both the periportal and perivenous zones (69).

**Figure 1:**
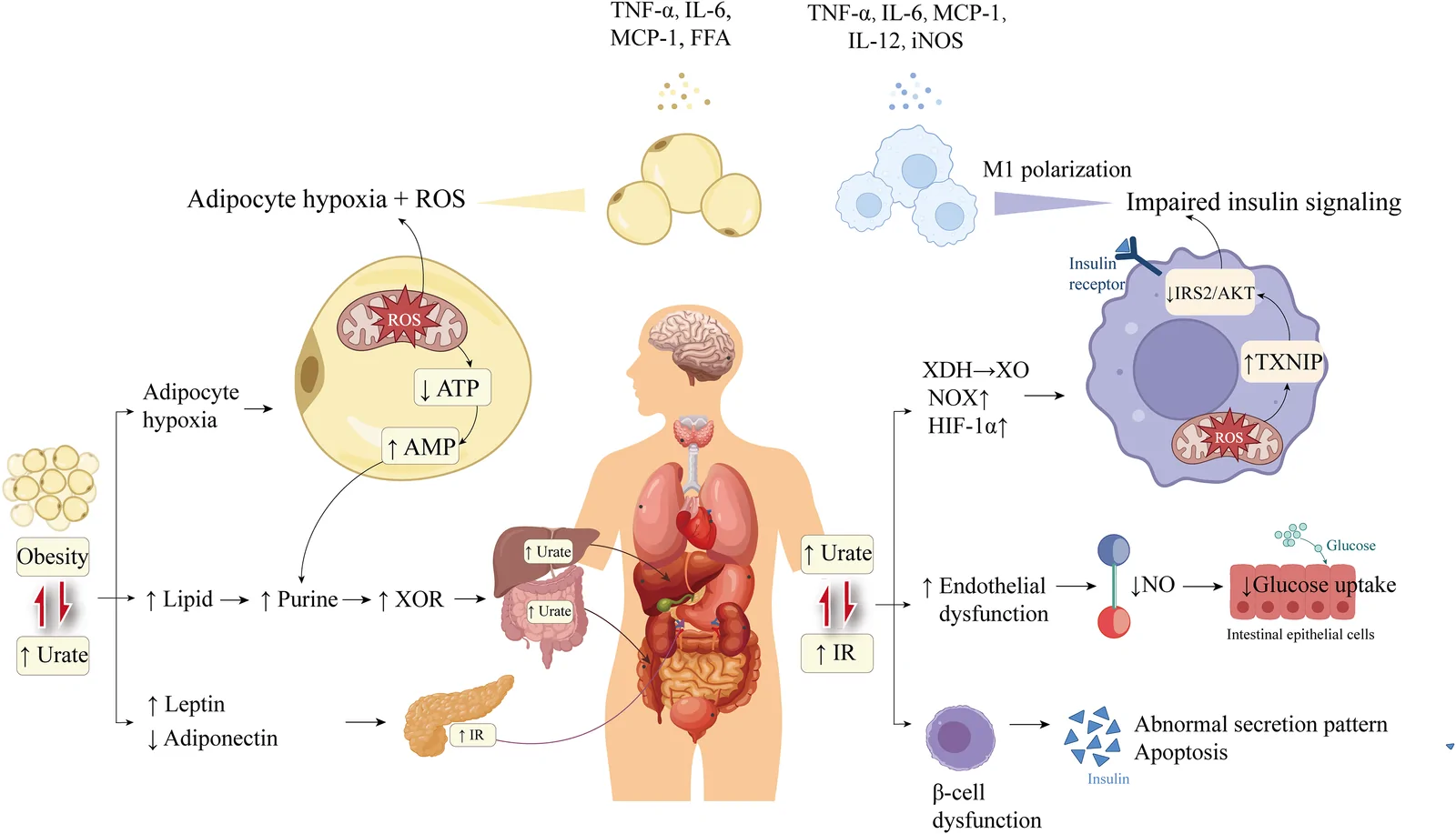
Core pathological pathways of metabolic disorders driven by hyperuricemia (HUA) in children. ATP, adenosine triphosphate; AMP, adenosine monophosphate; UA, uric acid; ROS, reactive oxygen species; FFA, free fatty acids; TNF-α, tumor necrosis factor-α; IL-6, interleukin-6; MCP-1, monocyte chemoattractant protein-1; IL-12, interleukin-12; iNOS, inducible nitric oxide synthase; XOR, xanthine oxidoreductase; XDH, xanthine dehydrogenase; XO, xanthine oxidase; TXNIP, thioredoxin-interacting protein; IRS2, insulin receptor substrate-2; AKT, protein kinase B; NOX, NADPH oxidase; HIF-1α, hypoxia-inducible factor-1α; NO, nitric oxide; IR, insulin resistance. The central anatomical human-body illustration was adapted from an iStock vector illustration by MicrovOne (Stock illustration ID: 1280413779) under the iStock Standard License. The liver, intestine, pancreas, and some cellular illustrations were adapted from BioRender under a BioRender Publication License.

Lifestyle interventions, including low-fructose diets and weight loss, have been demonstrated to reduce SUA levels and improve hepatic outcomes. Nonetheless, the significance of ULT in the management of juvenile MASLD is underexplored (70, 71). The core pathological pathways linking HUA to metabolic disorders in children are summarized in **Figure 1**.

### 5.5 Cardiovascular disease

#### 5.5.1 Hypertension

HUA in children is consistently associated with elevated blood pressure (BP) and increased cardiovascular risk. A meta-analysis found significant pooled correlations of SUA with systolic blood pressure (SBP) and diastolic blood pressure (DBP) in children and adolescents (61). In contrast, a nationally representative cross-sectional study of 1384 Taiwanese adolescents aged 14–19 years found that each 1 mg/dL increase in SUA was associated with a small but significant increase in DBP, but not SBP, after full adjustment (72). More recently, a population-based longitudinal cohort of 7525 Chinese children and adolescents suggested that UA partially mediated the relationship between BMI and BP (73). Additionally, higher SUA levels in childhood were associated with elevated BP in young adulthood. A cohort study involving 1,129 Japanese children aged 12–13 years, with an average follow-up duration of 8.6 years, revealed that young men with elevated BP exhibited higher baseline UA levels during childhood (3).

However, in a study involving 140 children with a mean age of 13.9 ± 2.6 years assessed for hypertension, Taner and colleagues found no independent association between UA levels and SBP after accounting for obesity and sex (74). A cross-sectional study examining the relationship between SUA levels and renal function parameters found no association between hypertension and elevated SUA in children with CKD (75). In children with impaired renal function, reduced urate excretion may become the main mechanism driving HUA. In this case, the strong confounding effect of renal insufficiency may mask the independent contribution of UA to hypertension observed in the general population. In addition, the weak relationship between BP and SUA in children may be related to sex hormones, growth, and other variables.

The role of UA in the pathogenesis of hypertension continues to be explored, despite some conflicting findings. Mechanistic evidence is derived mainly from animal models, *in vitro* studies, and limited adolescent clinical observations. In oxonic acid–induced rat models, mild HUA increased SBP without intrarenal urate-crystal deposition and was accompanied by activation of the renin–angiotensin system and reduced macula densa nitric oxide synthase 1 (NOS1) expression (76). Additional rat and *in vitro* data suggest that UA may promote renal microvascular remodeling through Ang II/angiotensin II type 1 receptor (AT1R)-related pathways (77). Higher SUA levels in adolescents have been confirmed to be associated with a higher Ang II/Ang- (1–7) ratio, rather than with Ang II alone (78). Gruskin and colleagues compared adolescents with primary hypertension to age-matched healthy controls with normal BP. The study found that within the hypertensive group, plasma renin activity (PRA) was higher in those with HUA than in normouricemic hypertensive adolescents (79). Experimental evidence suggests that elevated SUA can induce hypertension by activating the epithelial sodium channel (ENaC) (80). Childhood BP may be affected by various metabolic factors, including sex hormones and growth-related factors. Additional research is required to elucidate the changes in these relationships with age and to determine how other confounding or modifying factors influence these associations.

#### 5.5.2 Atherosclerosis and cardiovascular disease

Pediatric observational studies have associated elevated SUA with adverse cardiovascular risk profiles and subclinical vascular changes. Elevated SUA in children is associated with a clustering of early cardiovascular risk factors, including an atherogenic lipid profile, higher insulin levels, higher SBP and DBP, and lower IS (81). A cross-sectional study including 169 obese adolescents aged 10–16 years found a weak positive correlation between carotid intima–media thickness (cIMT) and SUA levels (82). Evidence summarized mainly from adult CVD and gout studies suggests that elevated UA is associated with pro-atherogenic pathways and vascular surrogate markers, including oxidative stress, endothelial dysfunction, platelet activation, inflammation, and arterial stiffness (83). Experimental evidence suggests that elevated UA promotes atherosclerotic progression by suppressing Nrf2–mediated autophagy and weakening the solute carrier family 7 member 11 (SLC7A11)/glutathione peroxidase 4 (GPX4) antioxidant axis, leading to enhanced macrophage ferroptosis and foam cell formation (84). Nevertheless, direct pediatric evidence supporting these mechanistic pathways remains limited. A pediatric mediation study suggests that SUA may contribute to early arterial stiffening indirectly through an IR–BP pathway, rather than acting as an independent determinant of vascular stiffness (85). Current evidence predominantly indicates an associative relationship between HUA and cardiovascular risk markers. However, there remains uncertainty about whether elevated UA levels function as an epiphenomenal indicator of metabolic dysregulation rather than as a direct pathogenic mediator.

### 5.6 Kidney diseases

The kidney both regulates and suffers from urate overload. Following glomerular filtration, the kidneys reabsorb around 90% of filtered urate and excrete 60-70% of the body’s total urate load (86).

Emerging data indicate that higher SUA was independently associated with CKD progression in children and adolescents. In the CKD in Children prospective cohort, SUA >7.5 mg/dL was independently associated with faster CKD progression in children and adolescents with CKD. This association corresponded to a roughly 1.61-fold increase in the risk of reaching a composite endpoint, defined as either a decline in GFR of over 30% or the initiation of kidney replacement therapy (KRT) (87). A longitudinal study of 153 children and adolescents with glomerular CKD and 540 with non-glomerular CKD found that higher baseline SUA was associated with faster eGFR loss (88). Additionally, in a cross-sectional study of 170 Chinese children with CKD, higher levels of blood urea nitrogen (BUN), a marker of renal dysfunction, were positively associated with SUA levels of ≥390 μmol/L (approximately 6.5 mg/dL) (75).

Mechanistically, mainly based on experimental and translational evidence, elevated UA reduces NO bioavailability and enhances oxidative stress (55). In endothelial and valvular models, UA has been shown to activate HIF-1α and thereby compromise endothelial barrier integrity and promote inflammatory cell infiltration (89). In parallel, experimental evidence from rat VSMCs suggests that UA activates the vascular renin–Ang system and increases Ang II production, thereby enhancing oxidative stress and promoting VSMC proliferation; these effects may contribute to increased glomerular capillary pressure (90, 91). UA further induces transcription of vasoactive mediators and nuclear factor kappa-B (NF-κB)–dependent inflammatory genes (92). Further mechanistic evidence showed that NF-κB activation suppresses aquaporin-2 (AQP2), aquaporin-3 (AQP3), and aquaporin-4 (AQP4) expression in collecting ducts, impairing urinary concentrating ability (93). Additionally, under physiological conditions, the PTEN-induced putative kinase 1 (PINK1)–Parkin pathway ubiquitinates and clears damaged mitochondria. In hyperuricemic nephropathy, autophagy dysfunction leads to mitochondrial accumulation and sustained ROS elevation, exacerbating renal cellular injury (94). Experimental evidence indicates that MSU crystals can activate the NLRP3 inflammasome. In a pediatric study, urinary IL-18/Cr was higher in patients with HUA, whereas KIM-1/Cr did not differ significantly between groups (95). Notably, this inflammatory cascade initially manifests as tubular epithelial inflammation rather than overt structural damage, highlighting a potentially reversible early disease window.

**Figure 2:**
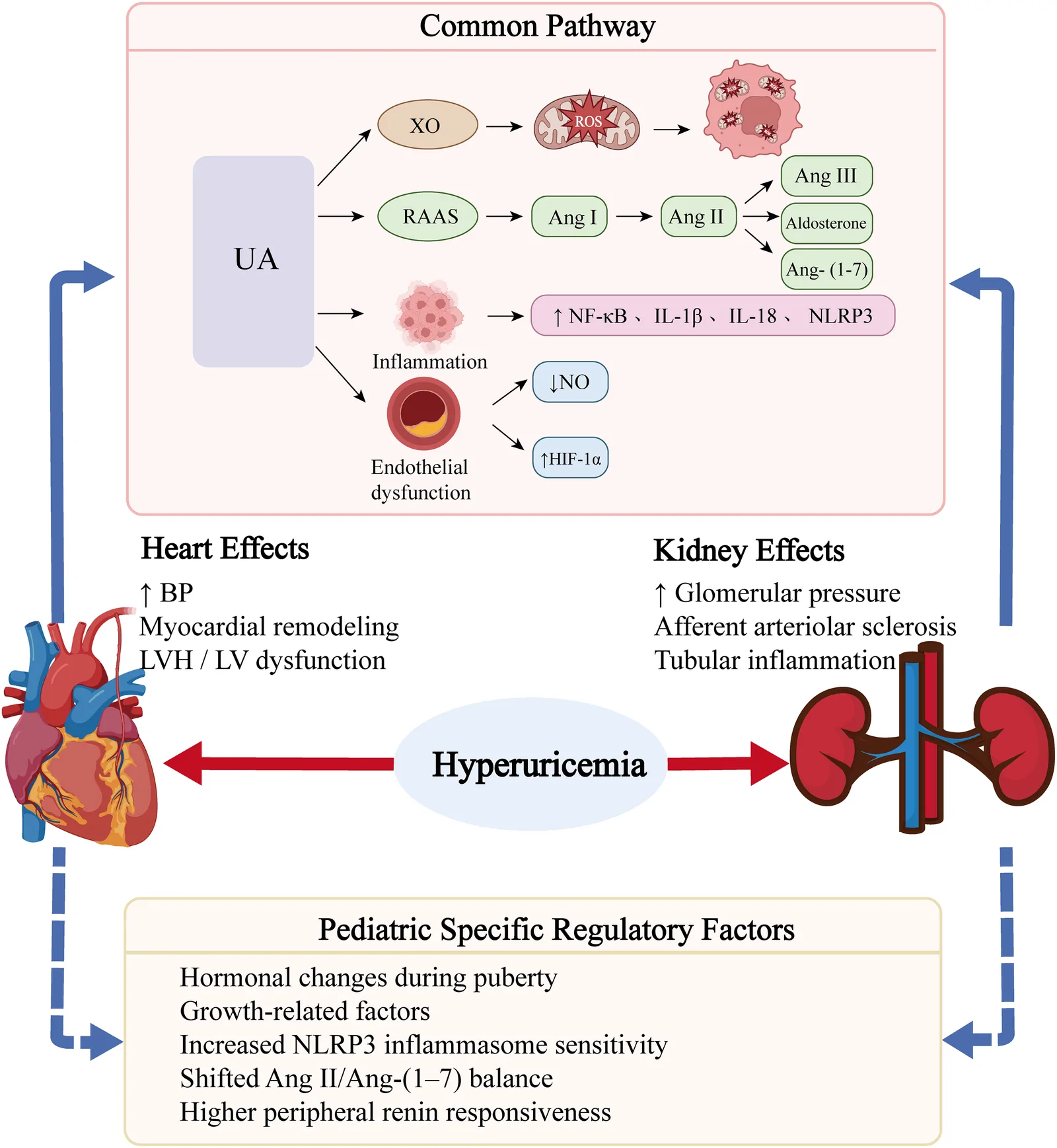
Pathophysiological pathways linking HUA to cardiorenal injury and pediatric-specific modifiers. UA, uric acid; RAAS, renin–angiotensin–aldosterone system; Ang I, angiotensin I; Ang II, angiotensin II; Ang III, angiotensin III; Ang-(1–7), angiotensin-(1–7); XO, xanthine oxidase; ROS, reactive oxygen species; NF-κB, nuclear factor kappa B; IL-1β, interleukin-1 beta; IL-18, interleukin-18; NLRP3, NOD-like receptor protein 3 inflammasome; NO, nitric oxide; HIF-1α, hypoxia-inducible factor-1 alpha; BP, blood pressure; LVH, left ventricular hypertrophy; LV, left ventricle. The heart and some cellular illustrations were adapted from BioRender under a BioRender Publication License.

Dynamic serum metabolomic profiling in children with IgA nephropathy implicated purine metabolism in disease progression. In the same study, HUA was associated with more severe pathological findings, including a higher proportion of Lee grade IV–V lesions and crescents, as well as poorer renal outcomes (96). Interestingly, the predictive value of SUA for kidney injury may vary by metabolic phenotype. A retrospective study of 396 obese children discovered that SUA levels were a significant predictor of kidney injury only in children with the metabolically healthy obese (MHO) phenotype, not in those with the metabolically unhealthy obese (MUO) phenotype (97). The key pathophysiological pathways linking HUA to cardiorenal injury, along with pediatric-specific modifiers, are illustrated in **Figure 2**.

## 6 Treatment of hyperuricemia

### 6.1 Lifestyle intervention

Lifestyle intervention serves as the foundation for HUA therapy in children and adolescents and is currently the most effective adjunct to ULT for the vast majority of pediatric patients. It should include a well-balanced diet with fewer energy-dense, sugar-rich, and fat-rich foods, increased daily physical activity, and behavioral therapy (98).

A limited number of clinical trials have examined lifestyle modification for HUA in children. A recent study in a population of obese children and adolescents found a reduction in UA levels in children who received a multifactorial lifestyle intervention and lost weight during the trial, but an increase in those who gained weight (99). In a cohort follow-up study of 276 patients with an average age of 10.6 years, Giussani et al. discovered that the higher the baseline UA levels, the worse the response to intervention (100). Pediatric observational evidence suggests that higher baseline SUA may attenuate the BP-lowering response to lifestyle intervention in children at increased cardiovascular risk (101). Although the effect of lifestyle intervention programs on obesity is promising, the influence on HUA needs to be studied further over a longer duration.

### 6.2 Nutrition recommendations

#### 6.2.1 Lifestyle and dietary factors

Nutritional adjustment is the most effective and safest intervention for reducing SUA in children. However, direct pediatric evidence demonstrating SUA reduction through dietary modification alone remains limited. Adult observational data suggest that the diet–HUA association may be partly mediated by obesity (102). Balanced dietary patterns such as the Mediterranean or Dietary Approaches to Stop Hypertension (DASH) diet—rich in vegetables, whole grains, legumes, and unsaturated fats—lower SUA by reducing oxidative and inflammatory burden (103, 104). In Chinese children and adolescents, moderate to high dietary choline consumption, particularly phosphatidylcholine and betaine, may reduce the risk of HUA by enhancing glomerular filtration function (105). Simple carbohydrates, notably fructose, increase UA synthesis through ATP breakdown, even though they do not contain purines. Adolescent observational studies have also shown that higher intake of fructose or sugar-sweetened beverages is associated with higher UA levels (106, 107). Although direct pediatric evidence on replacing sugar-sweetened beverages with coffee or tea is limited, caffeine intake itself was not associated with HUA or adverse cardiometabolic markers in a cross-sectional NHANES study of U.S. adolescents (108).

#### 6.2.2 Vitamins

Vitamins play key roles in growth, metabolism, and the maintenance of a robust immune system. The role of vitamins in UA metabolism is complex and varies by type. Vitamin D deficiency can activate human parathyroid glands and stimulate the release of parathyroid hormone (PTH), while SUA levels increase with elevated serum parathyroid hormone concentrations. Therefore, sufficient vitamin D levels appear to play a preventive role against the age-related increase in SUA. In a 2-year prospective cohort of metabolically healthy Chinese children and adolescents, each 10 nmol/L increment in 25-hydroxyvitamin D [25(OH)D] was associated with an 11% lower risk of HUA in children with overweight and a 7% lower risk in those with obesity (109). However, a cross-sectional study on children and adolescents conducted by Ma et al. revealed an inverted U-shaped, nonlinear relationship between serum 25(OH)D, 25-hydroxyvitamin D3 [25(OH)D3], and SUA levels with inflection points of 24.31 and 23.79 ng/mL (110). The dynamics of vitamin D absorption and metabolism, along with UA production and metabolism, may significantly differ in children and adolescents when compared to adults (111). Therefore, this difference underscores the need for age-specific studies.

Vitamin A, a fat-soluble essential vitamin, is involved in a wide range of physiological functions. In U.S. adolescents, cross-sectional NHANES data suggested a positive association between serum vitamin A indicators, especially retinol plus retinyl esters, and SUA (112). A cross-sectional study of 3,025 school-aged children and adolescents (aged 7–17 years) found that SUA was positively associated with vitamin A levels, and HUA was more prevalent in children and adolescents with higher serum vitamin A levels (113). These results indicate that an elevated vitamin A status might be linked to pediatric HUA, thereby underscoring the need for a prudent stance towards excessive vitamin A supplementation. Nevertheless, the causal relationship and the impact of interventions in this context still lack conclusive evidence.

Mechanistic and adult-based evidence suggests that vitamin C exerts uricosuric effects by inhibiting reabsorption of urate via transporters such as URAT1, solute carrier family 5 member 9 (SLC5A9) and solute carrier family 5 member 12 (SLC5A12) in the proximal renal tubule, thereby enhancing urate excretion (114). However, pediatric interventional evidence supporting vitamin C as a urate-lowering strategy remains limited. A clinical study of children with end-stage renal disease (ESRD) found that vitamin C treatment significantly lowered SUA, cholesterol, low-density lipoprotein cholesterol (LDL-C), and triglyceride levels (115). Furthermore, cross-sectional evidence from U.S. adolescents showed that higher serum vitamin C concentrations were inversely associated with SUA levels and HUA (112).

#### 6.2.3 Gut microbiota

The gastrointestinal tract plays a crucial role in urate excretion. Evidence from animal studies further suggests the involvement of the gut microbiota in purine and UA metabolism (116). Mechanistic evidence summarized in recent reviews suggests that the gut microbiota may promote intestinal UA elimination through enzymatic UA degradation and metabolite-mediated regulation of urate excretion. Certain gut microbiota, such as Lactobacillus and Pseudomonas, may contribute to UA degradation through uricolytic enzymes, while secreting short-chain fatty acids (SCFAs) produced by gut bacteria may modulate intestinal UA excretion (117, 118). However, pediatric evidence remains preliminary. A cross-sectional study involving 80 children showed that SUA levels positively correlated with genera Actinomyces, Morganella, and Streptococcus, and were negatively associated with the producers of SCFAs, such as Alistipes, Faecalibacterium, and Oscillospira, and the sulfidogenic bacteria Bilophila (119). However, it remains unclear how the dynamic changes in the pediatric gut microbiota with age affect UA metabolism due to the lack of large-scale cohort studies. Dietary changes are key to gut microbiota formation and alteration, and play a pivotal role in pharmacotherapy. Nevertheless, the mechanism by which fructose intake interacts with the gut microbiota remains unclear. Current research evidence mostly relies on animal experiments; the differences between humans and experimental animals (such as rats) limit the extrapolation of the results. Therefore, further clinical trials are required to clarify the negative impact of a high-sugar, high-fat diet on the microbial diversity of children.

### 6.3 Physical activity recommendations

Adult observational machine-learning evidence suggests that individuals with HUA may benefit from engaging in 1–7 h/week of PA and limiting sedentary time to <6 h/day to reduce gout risk (120). These findings may support general advice to reduce sedentary behavior and encourage age-appropriate moderate PA in pediatric HUA management. Regular moderate physical activity is recommended for pediatric HUA, but excessive training should be avoided. Kuo et al. reported a relatively high prevalence of HUA among Taiwanese adolescent athletes engaged in >10 h/week of moderate-to-vigorous physical activity, with HUA observed in 27.1% of males and 21.8% of females (121).

### 6.4 Pharmacologic treatment according to clinical indications

#### 6.4.1 Asymptomatic hyperuricemia

The current guidelines for asymptomatic hyperuricemia (AH) differ widely. Most European and American adult gout guidelines do not recommend initiating ULT for AH. Moreover, these guidelines are mainly targeted at adults and do not provide specific recommendations for AH in children. In adult gout-focused guidance, the 2020 American College of Rheumatology guideline conditionally advises against initiating pharmacological ULT for individuals with AH (122). This approach is primarily based on the lack of conclusive evidence supporting the benefits of lowering UA in these individuals, as well as the potential hazards and side effects of urate-lowering medications. According to the 2023 Chinese multidisciplinary expert consensus, pharmacological ULT should be considered in adults with AH when SUA levels exceed 540 μmol/L (approximately 9.1 mg/dL), or exceed 480 μmol/L (approximately 8.1 mg/dL) in patients with relevant comorbidities (123). According to the 2025 adolescent clinical practice consensus statement, patients with AH should receive lifestyle counseling and periodic monitoring. ULT may be considered when SUA persists at ≥420 μmol/L after 3–6 months of lifestyle intervention, increases to ≥540 μmol/L, or reaches ≥480 μmol/L in the presence of comorbidities, including obesity with metabolic abnormalities, CKD stage 2 or above, nephrolithiasis, or urate crystal deposition (98). The general clinical guidelines in Japan suggest that for AH, pharmacologic intervention may be cautiously considered when the SUA level is ≥8.0 mg/dL and there are relevant comorbidities, including kidney injury, urinary calculi, hypertension, ischemic heart disease, diabetes, or MetS. Furthermore, even in the absence of these comorbidities, drug therapy may also be considered when SUA levels are ≥9.0 mg/dL (124).

In general, the routine use of pharmacological ULT is not recommended for children with chronic AH. Adult guidelines should only be considered as indirect evidence. In cases where ULT is being considered, it should be limited to adolescents who demonstrate persistent and severe HUA despite lifestyle interventions, or those with high-risk comorbidities. This decision should be made based on an individualized assessment and under close, vigilant monitoring.

#### 6.4.2 Pediatric gout

Pediatric gout is uncommon and should be distinguished from asymptomatic HUA or subclinical MSU crystal deposition. However, chronic and persistent HUA in children may lead to MSU crystal deposition, which may subsequently progress to gout (125). In a large Japanese health insurance database including 696,277 individuals aged 0–18 years, the recorded prevalence of gout and AH was 0.040%, while the prevalence of gout alone was 0.007%. Notably, gouty arthritis was observed in 43.8% of pediatric patients diagnosed with gout (126). Because gout is rare in children, its occurrence should prompt evaluation for underlying contributors, including obesity, renal disease, inherited purine metabolism disorders, and genetic defects affecting urate handling.

The management of pediatric gout should distinguish between control of acute flares and long-term ULT. Compared with adults, adolescents with gout have been reported to present with higher serum urate levels, more frequent attacks, a greater number of affected sites, and higher pain scores (127). Accordingly, acute-phase management should focus on rapid suppression of inflammation and pain relief. Adult gout guidelines recommend colchicine, nonsteroidal anti-inflammatory drugs (NSAIDs), or glucocorticoids as first-line options for acute flares (122). However, these recommendations are mainly derived from adult trials and should not be directly extrapolated to children. A recent consensus statement on adolescent gout recommends early initiation of anti-inflammatory and analgesic treatment during acute attacks. Pharmacological options may include short-term oral glucocorticoids, NSAIDs, and colchicine. It is important to highlight that oral glucocorticoids should not be prescribed for durations exceeding 7 days (98). Direct pediatric evidence is limited. In a Chinese single-center cohort of 111 patients with juvenile gout, Zheng et al. reported that acute gout attacks were treated with NSAIDs and colchicine (128). However, the report did not define the specific regimens, doses, duration, or acute-treatment-related adverse events. The current evidence supporting the use of these medications primarily originates from studies on acute gout in adults (129), evidence from other pediatric inflammatory conditions such as systemic juvenile idiopathic arthritis (130), and data on colchicine toxicity (131). Among these, particular emphasis should be placed on the use of glucocorticoids and the toxicity issues associated with colchicine.

Because acute treatment only controls inflammation and does not eliminate the underlying urate burden, long-term ULT may be considered in adolescents with recurrent flares, tophi, or persistent symptomatic gout. This recommendation should be made cautiously, because no ULT has been broadly approved specifically for chronic pediatric gout across regulatory settings, and most use in pediatric gout remains off-label. In a Japanese health insurance claims database, febuxostat and allopurinol were the most commonly prescribed urate-lowering therapies among pediatric patients with gout or AH. Notably, among those with kidney disease, febuxostat was prescribed at a rate more than double that of allopurinol (132). Such prescribing patterns should not be interpreted as evidence of superior efficacy or safety.

#### 6.4.3 Uric acid nephrolithiasis

UA nephrolithiasis is a clinically important but relatively uncommon manifestation of pediatric HUA. Urinary UA saturation depends on the urinary UA concentration, urine pH, and urine volume, among which low urine pH is the major determinant of UA precipitation (133). Once UA stones are confirmed or strongly suspected, clinicians should systematically assess for hyperuricosuria, inherited disorders of purine metabolism, CKD, obesity-related acidic urine, dehydration, chronic diarrhea, and urinary tract anatomical abnormalities.

Pediatric UA nephrolithiasis requires a complete metabolic evaluation. A 24-hour urine collection should be analyzed for calcium, oxalate, UA, sodium, citrate, creatinine, urine volume, pH, and cystine (134). In infants and young children in whom reliable 24-hour urine collection is not feasible, random urine solute-to-creatinine ratios may be used instead. Importantly, in children, the interpretation of these results must take body weight, body surface area, and creatinine levels into account.

Available pediatric-specific urolithiasis literature supports a management strategy centered on acute complication control rather than serum urate lowering alone. Adequate hydration is essential. Urological evaluation or intervention should be considered in children with intractable pain, obstructive uropathy, associated infection or urosepsis, renal function deterioration, a solitary kidney, or failure of stone migration within an appropriate observation period (135). Pediatric evidence suggests that medical expulsive therapy with tamsulosin may facilitate spontaneous passage of selected distal ureteral stones (136). However, the available pediatric evidence is not specific to UA stones, and its use remains off-label.

Long-term treatment of pediatric UA nephrolithiasis is mainly based on increasing urine volume and alkalinizing the urine. Adequate fluid intake reduces urinary UA concentration. Although adults are usually advised to maintain a urine output of approximately 2.5 L/day, children should generally be encouraged to achieve an age- and weight-appropriate urine output. Pediatric urolithiasis references recommend maintaining a urine flow of at least 1.0 mL/kg/h (134, 135). Excessive intake of dietary animal protein may also increase purine intake, enhance UA production, and contribute to both uricosuria and urine acidification. Children with stones should avoid excessive protein intake, but the goal should still be to meet 100% of the recommended daily allowance to support normal growth and nutrition (134).

Potassium citrate is the preferred alkalinizing agent for pediatric UA stones, because sodium-containing alkali may increase urinary calcium excretion (134, 137). In infants and children, the initial dose of potassium citrate is 1–2 mEq/kg/day, with subsequent adjustment according to urine pH, urinary citrate, serum potassium, renal function, and gastrointestinal tolerability (138). The usual target urine pH for UA stones is 6.0–6.5 (135). Over-alkalinization should be avoided because it may increase the risk of calcium phosphate crystallization (134).

Xanthine oxidase inhibitors (XOIs), such as allopurinol, should not be routinely used in all children with UA stones. They may be considered when hyperuricosuria persists despite adequate hydration, dietary modification, and urinary alkalinization, or when UA overproduction is suspected, such as in certain inherited disorders of purine metabolism (135, 139). Published pediatric stone literature has described age-based allopurinol dosing, such as 50 mg/day in children younger than 10 years and 100 mg/day in older children (135). In contrast, more comprehensive pediatric stone management references suggest an allopurinol dosage range of 4–10 mg/kg/day, administered in divided doses, not exceeding a maximum of 300 mg per day (134). However, these dosing schemes are derived from pediatric stone reviews and clinical experience rather than randomized trials or a universally approved indication for pediatric UA nephrolithiasis.

#### 6.4.4 Inherited purine metabolism disorders and related metabolic diseases

Hereditary disorders of purine metabolism and related metabolic diseases often require disease-specific management. These conditions usually involve identifiable enzymatic or metabolic defects and may present early in life with HUA, hyperuricosuria, purine-related nephrolithiasis, gout, renal impairment, or neurological manifestations (140, 141). Therefore, treatment should be individualized according to the underlying disease, clinical phenotype, renal involvement, and risk of crystal-related complications.

In cases of hypoxanthine-guanine phosphoribosyltransferase 1 (HPRT1)-related HGPRT deficiency, an inborn error of purine metabolism results in overproduction of UA, along with motor dysfunction that mimics severe cerebral palsy, intellectual disability, and self-injurious behaviors (142). In a long-term mixed pediatric and adult cohort of patients with HPRT deficiency, allopurinol normalized SUA levels and markedly reduced urinary urate excretion, with an overall favorable safety profile (143). However, it increased urinary hypoxanthine and xanthine excretion. Therefore, careful dose adjustment is required to avoid excessive oxypurine accumulation and xanthine stone formation. The commonly reported allopurinol dose in this cohort was approximately 5 mg/kg/day, with a mean dose of about 6.4 mg/kg/day. However, allopurinol is considered an off-label medication for children with HPRT1 deficiency, and thus, its administration should be tailored on an individual basis. In addition, based on mixed pediatric–adult clinical evidence in HGPRT deficiency, allopurinol controls UA overproduction but is not expected to improve neurological or neurobehavioral manifestations. Neurological symptoms, dystonia, spasticity, dysphagia, and self-injurious behavior require multidisciplinary supportive care rather than intensification of ULT (143, 144).

For phosphoribosyl pyrophosphate synthetase 1 (PRPS1)-related PRPP synthetase superactivity, management is directed at reducing UA overproduction, maintaining adequate hydration, and preventing urinary UA crystallization (145). However, pediatric evidence remains limited and is largely derived from rare disease reports and expert-based practice.

APRT deficiency is characterized by excessive production and renal excretion of 2,8-dihydroxyadenine (2,8-DHA). It primarily causes nephrolithiasis and 2,8-DHA crystal nephropathy rather than typical HUA (146). This condition is important in the evaluation of pediatric radiolucent stones or crystal nephropathy because it can mimic UA stone disease and should be considered in the differential diagnosis of purine-related radiolucent stones (147). Reported allopurinol dosing is usually 5–10 mg/kg/day (148). However, these regimens have not been validated in pediatric trials and should not be interpreted as universal pediatric dosing recommendations.

In GSD I, HUA is a secondary manifestation of the underlying disorder of glucose metabolism rather than a primary purine enzyme defect. Based on pediatric-relevant, lifespan GeneReviews guidance and expert metabolic disease recommendations, management should first prioritize specialist-guided dietary and metabolic control. Allopurinol may be used to prevent or treat gout when dietary therapy does not fully normalize blood UA concentrations, particularly after puberty. Citrate supplementation may also be used to prevent urinary calculi or ameliorate nephrocalcinosis. Kidney transplantation may be required for ESKD, and combined liver–kidney transplantation may be considered when both metabolic correction and renal replacement are needed (149). These recommendations reflect expert guidance and clinical experience rather than pediatric randomized trial evidence.

#### 6.4.5 Chronic kidney disease-associated hyperuricemia

HUA is common in children with CKD, but whether pharmacological ULT can modify the progression of pediatric CKD remains uncertain. According to the 2024 Kidney Disease: Improving Global Outcomes (KDIGO) CKD guideline, patients with CKD and symptomatic HUA should receive urate-lowering intervention, particularly in the presence of gout flares or tophi. However, for patients with CKD and asymptomatic HUA, KDIGO recommends against the use of urate-lowering agents solely for the purpose of delaying CKD progression (150). Although these recommendations are for CKD patients of all ages, not just for the pediatric population, they provide an indirect reference for HUA associated with CKD in children.

Pediatric evidence remains limited and inconsistent. A study including 27 children with CKD who initiated allopurinol therapy and 81 untreated controls showed that allopurinol reduced SUA levels by approximately 15.9% compared with the control group. However, no significant between-group differences were observed in longitudinal changes in eGFR or proteinuria (151). A small prospective randomized study reported that 4 months of allopurinol treatment improved eGFR and reduced SUA, BP, and high-sensitivity C-reactive protein (hsCRP) levels in children with CKD, suggesting potential short-term renoprotective and anti-inflammatory benefits (152). However, this study was limited by its small sample size and short follow-up period, and large-scale pediatric trials remain lacking.

Therefore, routine pharmacological ULT is not currently supported for children with chronic CKD-associated asymptomatic HUA. Management should first focus on risk stratification and the identification of children at higher risk of CKD progression, such as those with rising SUA over time, worsening proteinuria, or rapid decline in eGFR. However, these features should be regarded as indicators for closer monitoring and individualized assessment rather than independent indications for initiating ULT.

#### 6.4.6 Tumor lysis syndrome-associated acute hyperuricemia

Tumor lysis syndrome (TLS) is a life-threatening metabolic disorder. TLS-related HUA manifests acutely as a direct result of the rapid lysis of tumor cells. This condition is concomitantly characterized by hyperkalemia, hyperphosphatemia, hypocalcemia, and an elevated risk of acute kidney injury (AKI) (153). The management of established or anticipated TLS includes early recognition, aggressive intravenous hydration, correction of electrolyte disturbances, and avoidance of nephrotoxic agents.

This indication differs from chronic pediatric HUA because the therapeutic goal is rapid prevention or reversal of UA-mediated AKI during tumor cytoreduction. Allopurinol reduces new UA production during TLS but has little direct effect on pre-existing SUA levels. In contrast, rasburicase rapidly lowers existing UA levels and reduces UA burden during tumor lysis, making it the preferred agent for high-risk patients. An expert consensus on TLS in adults and children recommends that ULT for malignancy-associated TLS in pediatric patients should be guided by risk stratification. Low-risk patients mainly require monitoring and hydration, and routine prophylactic ULT is generally unnecessary. For intermediate-risk patients, intensified hydration combined with allopurinol is recommended. For high-risk patients, intensified hydration combined with rasburicase is recommended. However, rasburicase is contraindicated in patients with glucose-6-phosphate dehydrogenase deficiency, in whom allopurinol should be used instead (154). Detailed drug-specific evidence, pediatric approval status, dosing considerations, and safety issues are summarized in Section 6.5.

### 6.5 Pharmacotherapy

The use of ULT in pediatric HUA is uncommon, and the real-world clinical application of ULTs in children has not been thoroughly explored. A summary of therapeutic drugs for treating HUA in children, with data from the key studies cited in this section (152, 155–164), is provided in **Supplementary Table 1**.

#### 6.5.1 Allopurinol

Allopurinol is a widely used XOI in clinical practice. However, its formally approved indications in pediatric patients remain highly limited, and most pediatric use is therefore off-label. According to U.S. prescribing information, allopurinol is indicated for adult and pediatric patients with leukemia, lymphoma, or solid tumor malignancies receiving cancer therapy expected to increase serum and urinary UA levels (165). Pediatric and adult TLS guidelines further support allopurinol as an antihyperuricemic option, particularly for intermediate-risk patients (166, 167). Off-label use has also been reported in selected pediatric patients with inherited disorders of purine metabolism (143), pediatric gout (132), UA nephrolithiasis (168), and CKD-associated HUA (152).

In a single-center retrospective pediatric cohort, Stiburkova et al. reported that allopurinol-based ULT was used in 73 children and adolescents with severe HUA, mainly those with inherited metabolic disorders, inherited kidney disease, CKD, or severe obesity. Empirical dosing began at 50 mg/day in young children and 100 mg/day in adolescents, with escalation to 200–300 mg/day in extremely obese adolescents. Following the treatment, SUA levels demonstrated a significant decline, dropping from an initial value of 556 ± 107 μmol/L to 350 ± 80 μmol/L (157). This dose range should be interpreted as a study-based or empirical regimen rather than a universally approved pediatric dose for chronic HUA or gout. In addition to its UA-lowering effect, allopurinol has also been demonstrated to have efficacy in reducing BP in hypertensive children with HUA. A well-designed double-blind, placebo-controlled, crossover trial involving 30 adolescents with newly diagnosed stage I primary hypertension and SUA levels ≥6 mg/dL found that allopurinol treatment resulted in significant reductions in both clinic and 24-hour ambulatory BP measurements compared to placebo (155). Additionally, a small prospective randomized open-label trial suggested that adding allopurinol to enalapril produced greater short-term reductions in systolic and diastolic BP than enalapril alone in adolescents with newly diagnosed stage 1 essential hypertension and HUA (156). A prospective randomized controlled trial showed that allopurinol treatment for 4 months can significantly increase the eGFR, reduce systolic and diastolic BP, and lower SUA and hsCRP levels (152). These small pediatric studies suggest possible short-term effects on BP and inflammatory or renal parameters, but they are insufficient to support routine allopurinol use for asymptomatic pediatric HUA or CKD progression prevention.

The 2025 British Society for Hematology guideline for TLS in adults and children with hematological malignancies recommends allopurinol as an option for low- or intermediate-risk TLS prophylaxis. In children, the recommended allopurinol dose is 100 mg/m² orally every 8 hours, started before definitive anticancer therapy (169). Before allopurinol is used for malignancy-associated HUA or TLS prophylaxis, baseline evaluation should include TLS risk assessment and pretreatment measurement of UA, renal function, and electrolytes, with monitoring frequency adapted to TLS risk (154, 167, 169).

However, it should be noted that allopurinol may cause rare but serious severe cutaneous adverse reactions (SCARs), such as Stevens-Johnson syndrome (SJS), particularly in the presence of the HLA-B*58:01 allele (170). HLA-B*58:01 screening should be considered before allopurinol initiation, particularly in populations with a high allele frequency or in patients at increased risk of SCARs. In the management of HUA in patients with purine enzymopathies, it is crucial to balance XO inhibition carefully. This balance aims to reduce SUA and urinary UA concentrations, thereby preventing the manifestation of gout and the formation of urate stones. However, excessive inhibition may lead to an accumulation of poorly soluble xanthine, posing the risk of xanthine urolithiasis. Therefore, achieving this equilibrium is essential for the effective treatment and long-term management of these patients.

#### 6.5.2 Febuxostat

Febuxostat is a non-purine XOI that exhibits greater selectivity and potency than allopurinol (171). In adult gout guidance, the European League Against Rheumatism (EULAR) states that febuxostat is generally positioned as an alternative to allopurinol when target SUA is not achieved or allopurinol is not tolerated (172). However, the safety and efficacy of febuxostat in children younger than 18 years have not been established, and its use is not currently recommended by relevant guidelines. Therefore, the use of febuxostat in children and adolescents with HUA should be regarded as off-label use. Consequently, dosing information for children and adolescents has not been standardized.

A Japanese claims-based study provides pediatric real-world evidence that febuxostat has been prescribed for children with gout or asymptomatic HUA, yet dosing remains empirical, largely based on adult regimens due to the absence of established pediatric guidelines (132). Iwama and colleagues conducted a pharmacokinetic analysis on a diverse population using a nonlinear mixed-effects model. The results indicate that febuxostat dosing in pediatric HUA patients (including gout) should be weight-adjusted (half the adult dose for those <40 kg and full adult dose for ≥40 kg), with no dose modification required for mild-to-moderate renal dysfunction (173). However, these pharmacokinetic findings require further validation in pediatric efficacy and safety studies. In Ito et al.’s prospective research, febuxostat doses varied by weight (5–60 mg/day), and 63.3% of pediatric patients reached the target UA levels with no major safety issues. However, one patient receiving a high dose experienced reversible ST-segment depression, suggesting potential cardiovascular concerns (158). The cardiovascular risks of febuxostat, which are now being debated in adults, are uncertain in children. There are insufficient long-term follow-up data for children with congenital cardiac disease or MetS. Further investigation is required. Zheng and colleagues conducted a retrospective cohort analysis to investigate the efficacy of febuxostat in patients with juvenile gout. Among 37 patients with juvenile gout who received febuxostat 40 mg once daily, the median SUA level decreased by 3.6 mg/dL after one month and remained stable after three months. The study also found high tolerability, with only four adverse events reported: three occurrences of increased transaminases and one of self-resolved rhabdomyolysis (128).

For adolescents receiving febuxostat, monitoring should include serial measurements of serum urate levels, baseline and periodic liver function tests, assessment of renal function, evaluation of cardiovascular history and symptoms, and surveillance for cutaneous reactions or hypersensitivity reactions (158, 174, 175). Concomitant use with azathioprine or mercaptopurine is contraindicated, as inhibition of XO may increase systemic exposure to these agents and lead to serious toxicity, including bone marrow suppression (176, 177). This contraindication is based on pharmacologic and prescribing-information evidence rather than pediatric-specific clinical trials. There is no published prospective, randomized clinical trial that compares the efficacy and safety of febuxostat to allopurinol in children with HUA. Current information is primarily based on small-sample or single-arm studies, which have limitations. To produce acceptable pediatric ULT, clinical trials on its efficacy and safety in the pediatric population are required.

#### 6.5.3 Rasburicase

Unlike allopurinol, which inhibits XO to prevent UA formation, rasburicase acts directly on pre-existing UA, catalyzing its conversion into the more soluble allantoin. This mechanism enables rapid UA clearance, making rasburicase particularly valuable in acute settings (178). Rasburicase is used to prevent and treat TLS-associated acute HUA in children with high-risk hematologic malignancies, particularly leukemia and lymphoma. Pediatric evidence includes prospective observational data, randomized open-label dose studies, and retrospective real-world experience showing rapid reductions in SUA levels and acceptable short-term tolerability (179–181). Rasburicase was first approved by the U.S. Food and Drug Administration in 2002 for the initial management of plasma UA levels in pediatric oncology patients at risk of tumor lysis–associated HUA (182).

The approved dose in both adults and children is 0.2 mg/kg once daily for up to 5 days, administered as a 30-minute intravenous infusion (169). However, a number of low-quality studies have investigated alternative strategies, including single weight-based dosing or fixed single-dose regimens. A small retrospective cohort study of children with severe HUA complicated by AKI observed that within less than 24 hours of rasburicase administration, UA levels decreased, accompanied by reductions in mean plasma creatinine and significant improvements in mean GFR (160). Another retrospective study revealed that rasburicase therapy was associated with shorter acute-phase hospitalization and better renal outcomes at 1-year follow-up. Notably, rasburicase also showed efficacy in children with typical hemolytic uremic syndrome (HUS)-associated AKI (163), suggesting potential off-label utility in non-oncologic pediatric HUA. However, these findings warrant confirmation in larger-scale studies.

Pediatric evidence suggests that rasburicase lowers UA more rapidly than allopurinol in selected settings. In a randomized multicenter trial of children with leukemia or lymphoma at high risk for TLS, rasburicase achieved significantly lower plasma UA exposure and more rapid UA control than oral allopurinol (164). Similar findings were reported in a small retrospective cohort of non-malignancy-associated acute HUA in pediatric cardiology patients (183). However, rasburicase is more expensive than allopurinol. To overcome this, various studies have investigated dose optimization methodologies. In a retrospective pediatric cohort restricted to patients weighing ≥30 kg, a fixed 6-mg dose of rasburicase achieved a 24-hour UA normalization rate similar to traditional weight-based dosing (184). A single-center retrospective cohort study compared low-dose rasburicase (≤0.1 mg/kg/day) with standard-dose rasburicase (>0.1–0.2 mg/kg/day) in 37 critically ill children with hematological malignancies. No statistically significant differences were observed between the two groups in UA normalization, serious complications, renal replacement therapy, short-term mortality, or ICU length of stay (162). These findings indicate that lower-than-recommended doses may be a more cost-effective alternative for critically ill children, especially in regions with limited healthcare resources. However, due to the small sample size, retrospective study design, and baseline differences in age and body weight, further prospective studies are needed to confirm the effectiveness of the fixed low-dose strategy.

Rasburicase is generally well tolerated in pediatric populations, with infrequent and mild side effects. In a large multicenter compassionate-use study of 1069 patients, including 682 children, possible drug-related adverse events were uncommon and mostly mild; however, rare serious events, including hypersensitivity reactions, hemolytic anemia, methemoglobinemia, and anaphylaxis, were reported (185). In pediatric cancer trials summarized in a Cochrane review, reported adverse events of urate oxidase included hypersensitivity, hemolysis, and anemia (186). Rasburicase is contraindicated in patients with glucose-6-phosphate dehydrogenase (G6PD) deficiency because of the risk of acute hemolytic anemia and possible methemoglobinemia (187). This recommendation applies to neonates, children, and adults. Monitoring during rasburicase therapy should include serial serum urate measurements, renal function, electrolytes, and clinical surveillance for hypersensitivity reactions, hemolysis, and methemoglobinemia (188).

#### 6.5.4 Urate transporter 1 inhibitors

Urate transporter 1 (URAT1) is a key urate transporter expressed on the apical membrane of proximal tubular cells, where it mediates the reabsorption of urate from the tubular lumen and regulates systemic urate homeostasis (189). Pharmacologic inhibition of URAT1 enhances renal urate excretion and consequently lowers circulating SUA levels. Several non-selective urate transporter inhibitors, including probenecid, benzbromarone, sulfinpyrazone, and lesinurad, have been clinically used as uricosurics. Benzbromarone exerts potent inhibitory activity against URAT1- and glucose transporter 9 (GLUT9) urate transport, but its clinical utility is limited by hepatotoxicity (190, 191). Dotinurad, a phenolic derivative rationally designed to minimize hepatotoxic risk, exhibits greater selectivity toward URAT1 and fewer off-target effects, and has been approved in Japan for the treatment of gout (192–194). Lesinurad was the most extensively studied URAT1 inhibitor and demonstrated robust SUA-lowering efficacy when co-administered with XOIs. However, its clinical utility was constrained by renal safety concerns, including serum creatinine elevations and a boxed warning for acute renal failure (195). Verinurad, developed as a higher-affinity and more selective next-generation analogue, has shown enhanced URAT1 activity, favorable tolerability, and synergistic efficacy when combined with febuxostat in Phase II trials (196). SHR4640 is another emerging URAT1 inhibitor, which demonstrated marked SUA reduction and acceptable safety in Chinese patients with HUA with or without gout (197).

Notably, clinical evidence supporting URAT1 inhibitor use in children and adolescents remains extremely limited, and no major guidelines or product labels currently recommend URAT1-targeting uricosurics in pediatric populations.

## 7 Conclusion

Recent advances have transformed our understanding of HUA in children. Unlike adults, pediatric urate homeostasis exhibits pronounced age-, sex-, and puberty-dependent variation, underscoring the need for standardized and internationally validated diagnostic criteria for pediatric HUA. Genetic determinants, including ABCG2 dysfunction, gene-based ADTKD subtypes, and inherited purine metabolism disorders, play an important role in early-onset disease. Secondary HUA is also relatively common in children, typically driven by metabolic stress and emerging cardio-renal risk profiles. Modifiable factors, including obesity, IR, micronutrient status, lifestyle patterns, and alterations in the gut microbiome, further shape urate metabolism and represent actionable intervention targets.

Therapeutic evidence in children remains limited. Pharmacological intervention ought to be implemented based on specific clinical indications, rather than being solely directed by SUA levels. Separate and tailored considerations are warranted for distinct clinical scenarios, including AH, pediatric gout, UA nephrolithiasis, inherited disorders of purine metabolism, symptomatic HUA associated with CKD, and acute HUA linked to TLS. Selective URAT1 inhibitors and microbiome-based strategies offer promising avenues, yet pediatric-specific clinical data are largely absent. Achieving consensus on diagnostic thresholds, therapeutic approaches, evidence gaps, and long-term outcomes is essential for altering the natural history of pediatric HUA and mitigating its cardiovascular, metabolic, and renal consequences throughout childhood and adolescence. Chronic asymptomatic pediatric HUA remains a major evidence gap, and management should be cautious and individualized.
