# Supplementary material for: Pediatric hyperuricemia: genetic basis, metabolic mechanisms, and clinical implications
Source: Front Endocrinol (Lausanne). 2026 Aug 10;17:1841803. doi: 10.3389/fendo.2026.1841803 (PMC13501462; doi:10.3389/fendo.2026.1841803)
Supplement: Supplementary file 1 [file Table1.docx]

Supplementary Table 1. Research summary of therapeutic drugs for treating HUA in children.

| Medication class, mechanism of action | Study design and sample size (ref. no.) | Age (years) | Agent dose/route | Comparator agent(s) | Duration | Effect on UA | Other effects | special considerations |
| --- | --- | --- | --- | --- | --- | --- | --- | --- |
| Allopurinol: Inhibits xanthine oxidase; reduces the production of UA. | RCT, n=30 | 11–17 | Allopurinol 200 mg/day, PO | Placebo | 10 weeks | Allopurinol, SUA decreased from 6.9 mg/dL to 4.2 mg/dL, a reduction of 2.7 mg/dL (p < 0.001). Placebo, SUA increased from 6.2 mg/dL to 6.4 mg/dL (p = 0.04). | Blood pressure decreases, renin activity declines, and the systemic vascular resistance index drops. | No drug-related adverse reactions. |
|  | Open-label RCT, n=52 | 12–19 | Allopurinol 5 mg/kg/day, PO Enalapril 0.15 mg/kg/day, PO | Enalapril | 8 weeks | Combined therapy, UA decreased from a baseline of 6.8 mg/dL to 5.1 mg/dL, a reduction of -1.70 mg/dL (p = 0.002). Enalapril, UA reduction of -0.51 mg/dL (p-value not reported). | Blood pressure decreases | No drug-related adverse reactions. |
|  | RCT, n=70 | 3–15 | Allopurinol 5 mg/kg/day, PO | Blank control | 4 months | Allopurinol, UA decreased from 6.7 ± 1.2 mg/dL to 5.2 ± 1.6 mg/dL (p < 0.01). | Improvement in renal function, reduction in blood pressure, and decline in inflammatory markers. | No drug-related adverse reactions. |
|  | Retrospective analysis, n=13,890 | 0–18 | Allopurinol Children: 50 mg; adolescents: 100 mg; extremely obese adolescents: 200–300 mg/day, PO. | None | Unspecified | Allopurinol, UA decreased from 9.35 ± 1.80 mg/dL to 5.88 ± 1.35 mg/dL. | Blood pressure decreases | No drug-related adverse reactions. |
| Febuxostat: Inhibits xanthine oxidase; reduces the production of UA. | retrospective analysis, n=45 | 5–15 | Febuxostat 10 mg/day, PO Allopurinol 300 mg/m²/day, PO | Allopurinol | 7 days | Febuxostat, UA decreased from 6.6 ± 3.8 mg/dL to 4.5 ± 2.8 mg/dL (p < 0.001). Allopurinol, UA decreased from 5.9 ± 2.1 mg/dL to 3.5 ± 1.1 mg/dL (p < 0.001). Inter-group comparison: The overall reduction rate was - 34.4% for febuxostat vs. - 42.1% for allopurinol (p = 0.16). | TLS occurrence | No drug-related adverse reactions. |
|  | Phase 2, single-arm, open-label, multicentre study, n=30 | 8–18 | Stepwise titration (5 - 60 mg, stratified by body weight) | None | 52 weeks | Febuxostat, UA decreased from 9.01 ± 1.23 mg/dL to 5.55 ± 0.87 mg/dL at Week 26 (a reduction of 37.08%) | None | Mild electrocardiogram abnormalities (incomplete right bundle branch block, ST-segment depression) |
| Rasburicase: Recombinant uricase drugs; directly decompose UA. | Retrospective study, n=15 | 0–18 | Rasburicase 0.15 mg/kg, IV | None | 24 hours | Rasburicase, UA decreased from 13.1 ± 2.19 mg/dL → 0.76 ± 0.62 mg/dL (p < 0.001) | Improvement in renal function, changes in electrolytes, and a decrease in urea. | No drug-related adverse reactions. |
|  | Phase IV prospective interventional study, n=47 | 2–18 | Rasburicase 0.02 mg/kg, IV | None | 1-5 days | Rasburicase, a maximum average reduction of 96.9% in UA. | Accompanied by chemotherapy, a decrease in serum potassium, an increase in ALT/AST, and a decrease in WBC. | Fever, hypokalemia, elevated ALT, elevated AST, decreased WBC, abdominal pain, nausea, vomiting, and anaphylactic shock. |
|  | retrospective analysis, n=37 | ＜18 | Standard dose group 0.11–0.20 mg/kg/day, IV Low dose group 0.04–0.10 mg/kg/day, IV | Inter-group comparison | 12h、24h、36–72h | Standard dose group，100% of UA levels reached the target within 12 hours. Low-dose group, 75% of UA levels reached the target within 12 hours (P = 0.083). | TLS occurs, and renal function recovers. | Allergic reactions, TLS (Tumor Lysis Syndrome), AKI (Acute Kidney Injury), Respiratory failure, DIC (Disseminated Intravascular Coagulation), Heart failure |
|  | retrospective analysis, n=42 | 1.9-4.4 | Rasburicase 0.10 mg/kg, IV | Standard supportive treatment | 1 year | Rasburicase group, the median duration of HUA was 36 hours. Control group, the median duration of HUA was 120 hours (p < 0.001). | Improvement of renal function | No drug-related adverse reactions. |
|  | Randomized, open-label, multicenter, controlled trial, n = 52 | 0.3-17 | Rasburicase 0.20 mg/kg, IV Allopurinol 300 mg/m² (or 10 mg/kg), PO | Allopurinol | 5-7 days | Four hours after the first dose of rasburicase, the uric acid level decreased by 86% (vs 12% in the allopurinol group, p < 0.0001). | Creatinine change | Hemolysis |

HUA, hyperuricemia; SUA, serum uric acid; CKD, chronic kidney disease; AKI, acute kidney injury; MetS, metabolic syndrome; IR, insulin resistance; HOMA-IR, homeostasis model assessment of insulin resistance; hs-CRP, high-sensitivity C-reactive protein; eGFR, estimated glomerular filtration rate; SBP, systolic blood pressure; DBP, diastolic blood pressure; cIMT, carotid intima–media thickness; BMI, body mass index; TG, triglycerides; HDL-C, high-density lipoprotein cholesterol.
